# A Gigantic Anal Mass: Buschke–Löwenstein Tumor in a Patient with Controlled HIV Infection with Fatal Outcome

**DOI:** 10.1155/2018/7267213

**Published:** 2018-04-01

**Authors:** Sanjog Bastola, Alexandra Halalau, Ojbindra Kc, Anju Adhikari

**Affiliations:** Department of Internal Medicine, William Beaumont Hospital, Royal Oak, MI, USA

## Abstract

Buschke–Löwenstein tumor of anorectal and perianal area is a rare but highly aggressive tumor, frequently associated with human papillomavirus (HPV) types 6 and 11. It often grows over years in immunocompetent patients and can be highly destructive to local tissue. We present a case of a 61-year-old male with HIV infection who presented with worsening pain and swelling in the anorectal area for one-year duration. Exam revealed a 15 × 10 cm mass in the anorectal area with multiple sinuses and fistulas. MRI revealed extension of the mass through pelvic structures. Biopsy showed squamous epithelium with koilocytes and histochemistry positive for P16, suggestive of HPV infection. Biopsy was negative for malignant transformation. He was not a candidate for surgery or radiation due to extensive infiltration of deeper structures and multiple fistulas. He refused interferon therapy, and diverting colostomy was placed for palliation. He presented two months later with overwhelming sepsis and died despite maximal medical therapy.

## 1. Introduction

Buschke–Löwenstein tumor, often called giant condyloma accuminatum, is considered by some authors as intermediate between condyloma and squamous cell carcinoma [1]. Histologically, the tumor appears benign with papillomatosis, epithelial hyperplasia, and koilocytosis, but clinically it can behave aggressively with extensive infiltration. Typically, it is slow growing in immunocompetent individuals, but it can grow rapidly in immunocompromised individuals [2]. Focally, these tumors can transform into invasive carcinoma; hence, early diagnosis and treatment is crucial [1]. Common treatment approach includes radical surgical resection with tumor-free resection margins. Prophylactic HPV vaccination has been shown to reduce HPV6/11 infection and anogenital condylomata and thus is expected to prevent this tumor [2].

## 2. Case Description

**Figure 1:**
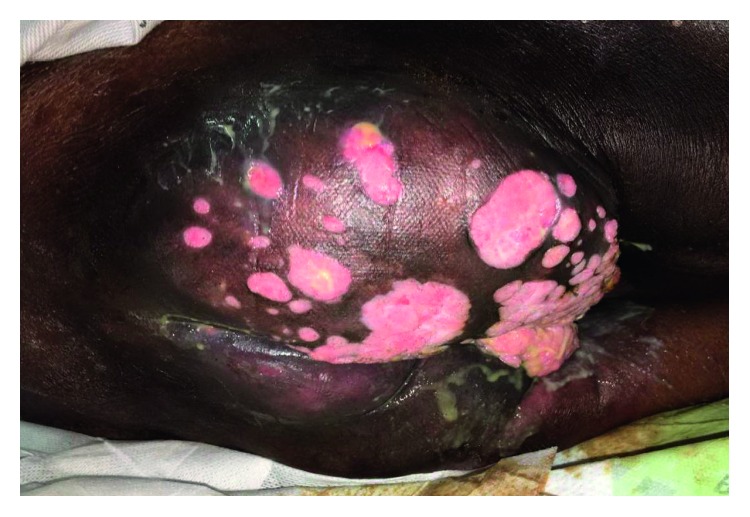
Cauliflower-like fungating mass with foul-smelling purulent discharge.

**Figure 2:**
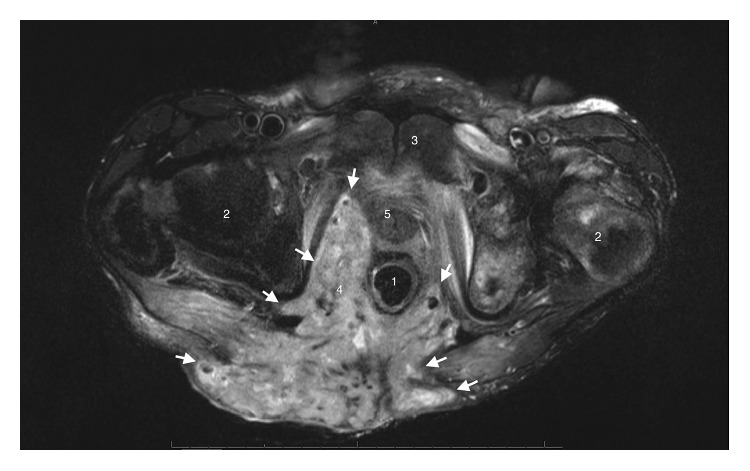
MRI of the pelvis shows extensive infiltration of the mass (indicated by solid arrow) into pelvic structures. (1) Rectum, (2) femoral head, (3) symphysis pubis, (4) ischiorectal fossa, and (5) urethra.

A 61-year-old MSM with a history of well-controlled HIV infection presented with a foul-smelling mass and worsening pain in the anorectal area for about a year. On presentation, he was septic, with a temperature of 101°F, blood pressure of 90/60 mmHg, HR of 105 beats/min, and an elevated white blood cells count of 12,000 cells/mm^3^ with high neutrophils of 9800 cells/mm^3^. His HIV viral load was <20 copies/ml, and his CD4 count was 480 cells/*μ*L. His HIV treatment regimen included lopinavir-ritonavir, raltegravir, and saquinavir which he was tolerating well. Physical examination revealed an approximately 15 cm × 10 cm fungating mass with multiple sinuses and fistulas involving entire right buttock and perineum along with a very foul-smelling purulent discharge (Figure 1). MRI of the pelvis revealed extensive necrotic tumor extending to the right pelvic sidewall including ischium, ischiorectal fossa, perineum, insertion of corpus cavernosum, scrotal base, and right gluteal area, with involvement of the subcutaneous tissues (Figure 2 and supplementary materials (available here)). Biopsy showed fragments of squamous epithelium with koilocytes and positive P16 staining by histochemistry consistent with HPV infection. Ki-67 staining was positive only in lower one-third of epithelium, indicating no high-grade dysplasia. ERG staining for endothelium was negative, suggesting no lymphovascular invasion. Focal atypical features were present in the lamina propria, but there was no definite evidence of invasive carcinoma. Underlying stroma showed marked inflammation with many plasma cells. Warthin–Starry stain for spirochetes was negative. Brown and Brenn stain for bacteria was negative and so was GMS staining for fungus. Biopsy was also obtained from the lesion in ischium which was negative for cytokeratin immunostain (AE1/AE3) and showed reactive changes. HPV viral typing was not performed. A diagnosis of Buschke–Löwenstein tumor was made due to the size of the mass and histological findings consistent with condyloma. The mass was deemed unresectable due to extensive local infiltration. The patient was offered systemic interferon therapy which he refused. He had multiple fistulas which excluded him from being a candidate for radiation therapy. Diverting colostomy was placed for palliation, and he was discharged home. He presented to the emergency department two months later for sepsis from secondary infection of the tumor and passed away despite optimal medical treatment in the intensive care unit.

## 3. Discussion

We presented a case of rapidly growing gigantic perianal condyloma in a controlled HIV-infected patient that was deemed unresectable at the time of presentation and ultimately led to the patient's demise secondary to septic shock.

Typically, this tumor presents as a slow-growing cauliflower-like mass in genital or anorectal region with slow infiltration into deeper tissues [3, 4]. It often starts from long-standing condylomata and can reach up to 10–15 cm as in our patient. Evolution period ranged from 2.8 to 9.6 years, as reported in a systematic review done by Chu et al. which included 42 BLT cases [5]; however, our patient had rapid growth in a year. Males are more commonly affected with male-to-female ratio of 2.7 :  1 [2]. When present in anorectal region, it is often associated with fistulas, anal stenosis, and abscesses [4]. Bacterial superinfection is common and is associated with very foul smell [3]. This tumor most often occurs in immunocompromised patients; however, it had a very aggressive course in our patient despite being virologically controlled and immune-reconstituted. Reported risk factors include anal receptive sex, HIV positive, immunosuppression, chronic irritation, and poor personal hygiene. Although low-risk HPV6/11 appears central to pathogenesis of this neoplasm, it is unknown what causes transformation of benign condylomata into locally invasive Buschke–Löwenstein tumor.

Gold standard for treatment is surgical resection with wide tumor-free surgical margins. It is essential as it has high rate of recurrence (66%) and malignant transformation (56%) [5]. Patients with extensive lesion, sinuses, and fistulas may require diverting colostomy and in severe cases may not be a surgical candidate [1]. Other treatment modalities include cryotherapy, photodynamic therapy, topical treatment with podophyllin, 5-fluorouracil, and systemic interferon. These treatment modalities are less effective and associated with higher rates of recurrence compared to surgical resection. Grodner et al. reported a case of Buschke–Löwenstein tumor that resolved with immune restoration with HAART and highlights the role of immunosuppression in pathogenesis [6]. In some cases, the tumor worsened with HAART initiation despite immune restoration [7].

Prognosis depends on the size of tumor on presentation and factors like immunosuppression and secondary infection. Immunosuppressed patients are at higher risk for malignant transformation with 40–60% patients having invasive squamous cell carcinoma [3]. Although this is typically a slow-growing tumor, some patients can have extremely aggressive clinical course with fatal outcome [8]. Rapid progression was seen in our patient despite having well-controlled HIV infection. We could not find other reported cases of rapid growth in well-controlled HIV as in our patient. This suggests that good viroimmunological control may be insufficient for controlling the HPV infections.

Prophylactic vaccination against HPV has been shown to reduce the incidence of HPV-associated HPV infections including HPV6/11 and thus is expected to also prevent Buschke–Löwenstein tumors.

## Supplementary Materials

Supplementary Materials Figure 1: MRI of pelvis shows an extensive mass measuring 10.4 cm × 15.1 cm (solid arrow) infiltrating pelvic structures.
