# Supplementary material for: A Gigantic Anal Mass: Buschke–Löwenstein Tumor in a Patient with Controlled HIV Infection with Fatal Outcome
Source: Case Rep Infect Dis. 2018 Apr 1;2018:7267213. doi: 10.1155/2018/7267213 (PMC5902119; doi:10.1155/2018/7267213)
Supplement: Supplementary Materials — Figure 1: MRI of pelvis shows an extensive mass measuring 10.4 cm × 15.1 cm (solid arrow) infiltrating pelvic structures. [file 7267213.f1.docx]

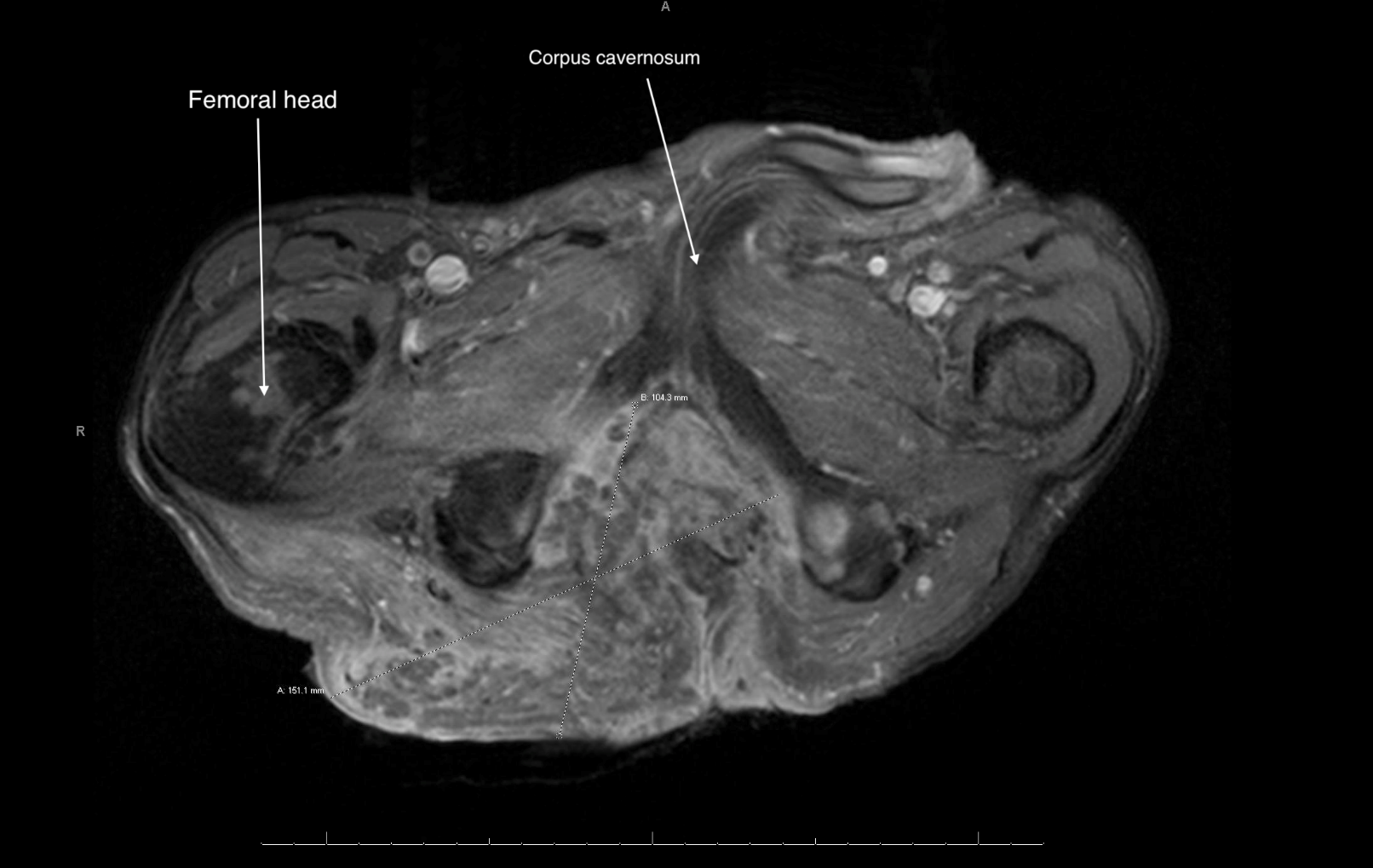


Figure 1: MRI of pelvis shows extensive mass measuring 10.4 cm x 15.1 cm (solid arrow) infiltrating pelvic structures.
